# CAR-T Cells Immunotherapies for the Treatment of Acute Myeloid Leukemia—Recent Advances

**DOI:** 10.3390/cancers15112944

**Published:** 2023-05-27

**Authors:** Julia Zarychta, Adrian Kowalczyk, Milena Krawczyk, Monika Lejman, Joanna Zawitkowska

**Affiliations:** 1 Student Scientific Society of Department of Pediatric Hematology, Oncology and Transplantology, Medical University, 20-093 Lublin, Poland; julia.zarychta99@gmail.com (J.Z.); adriankowalczyk31@gmail.com (A.K.); milenakrawczyk77@gmail.com (M.K.); 2 Independent Laboratory of Genetic Diagnostics, Medical University of Lublin, 20-093 Lublin, Poland; monika.lejman@umlub.pl; 3 Department of Pediatric Hematology, Oncology and Transplantology, Medical University, 20-093 Lublin, Poland

**Keywords:** CAR-T, AML, CD33, CD123, FLT3, CLL-1

## Abstract

**Simple Summary:**

Despite intensive standard treatment, acute myeloid leukemia (AML) continues to be associated with a poor prognosis due to treatment resistance and a high risk of relapse. For this reason, it is necessary to develop new therapeutic strategies, which, when used in combination with standard treatment, can increase the chances of patient survival. One of them might be the use of CAR-T cells. In the following review, we present the latest reports on the use of anti-CD33, -CD123, -FLT3 or -CLL-1 CAR-T cells in (pre-)clinical trials, as well as the emerging challenges of the proposed therapy. The use of CAR-T cells in common clinical practice in the treatment of AML will only be possible after further research, the focus of which should be the myeloablative effect of CAR-T cells, the eradication of cells in the immunosuppressive environment of the tumor, and their life span in the body.

**Abstract:**

In order to increase the effectiveness of cancer therapies and extend the long-term survival of patients, more and more often, in addition to standard treatment, oncological patients receive also targeted therapy, i.e., CAR-T cells. These cells express a chimeric receptor (CAR) that specifically binds an antigen present on tumor cells, resulting in tumor cell lysis. The use of CAR-T cells in the therapy of relapsed and refractory B-type acute lymphoblastic leukemia (ALL) resulted in complete remission in many patients, which prompted researchers to conduct tests on the use of CAR-T cells in the treatment of other hematological malignancies, including acute myeloid leukemia (AML). AML is associated with a poorer prognosis compared to ALL due to a higher risk of relapse caused by the development of resistance to standard treatment. The 5-year relative survival rate in AML patients was estimated at 31.7%. The objective of the following review is to present the mechanism of action of CAR-T cells, and discuss the latest findings on the results of anti-CD33, -CD123, -FLT3 and -CLL-1 CAR-T cell therapy, the emerging challenges as well as the prospects for the future.

## 1. Introduction

The dual role of the immune system in the process of carcinogenesis is reflected in the hypothesis of immunoediting. On the one hand, the immune system can completely eradicate a tumor from an immunocompetent organism; and on the other hand, it can promote its progression by selecting the tumor cells best suited to overcome the host’s immunocompetent immune system. Tumor immunoediting proceeds in three phases: elimination, equilibrium, and escape [1,2]. In the elimination phase, an immune response is initiated against the tumor cells in order to eliminate them before the tumor becomes clinically visible. If the immune system does not destroy all cancer cells, the next stage of the immunoediting process begins, which can take years or decades. In the equilibrium phase, the immune system keeps the remaining cancer cells functionally dormant, preventing their further expansion. When cancer cells, as a result of selection pressure, develop mechanisms that allow them to evade the host’s immune response, the escape phase begins and the cancer becomes clinically visible [1,2,3,4]. Understanding the relationship between the immune system and cancer development has contributed to the progress of immunotherapy, the aim of which is to stimulate and increase the patient’s immune response against cancer cells with a view to eliminating them completely or maintaining them in the equilibrium phase [3,4].

**Figure 1:**
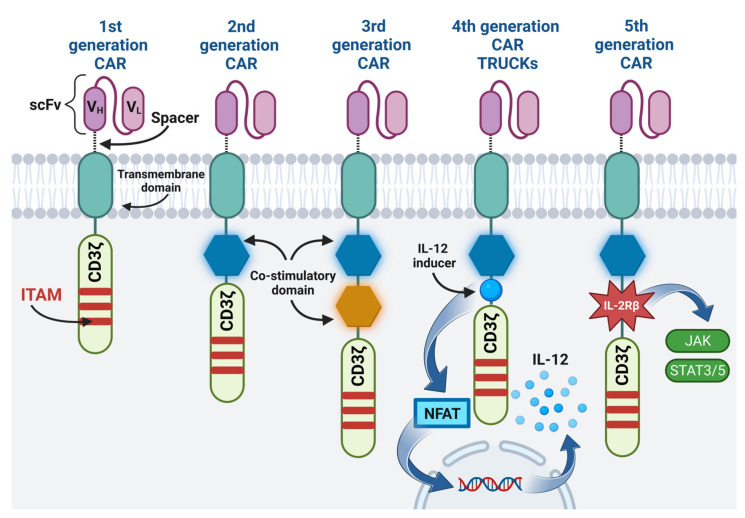
The structure of different CAR generations: The first generation contains only CD3ζ cytoplasmic domain with three immunoreceptor tyrosine-based activation motifs (ITAMs). The co-stimulatory domain is added in the second generation. The third generation contains two co-stimulatory domains. The fourth generation, apart from one co-stimulatory domain, additionally contains a transcription factor that brings about inflammatory cytokine production. The fifth generation, in addition to one co-stimulatory domain, contains IL-2Rβ, which triggers off JAK/STAT pathway activation. Image created with biorender.com (accessed on 22 April 2023). CAR—chimeric antigen receptor, scFv—single-chain variable fragment, V_H_—heavy chain variable segment, V_L_—light chain variable segment, CD3ζ—CD3ζ signaling domain, ITAM—immunoreceptor tyrosine-based activation motif, IL-12—interleukin 12, NFAT—nuclear factor of activated T cells, IL-2Rβ—interleukin 2 receptor subunit beta, JAK—janus kinase, STAT3/5—signal transducer and activator of transcription 3/5.

One example of immunotherapy is the adoption cell therapy, which involves the administration of immune cells with direct anti-cancer activity to a cancer patient [5]. Thanks to genetic engineering methods, T cells, previously isolated from the patient’s circulation, were obtained, expressing the chimeric antigen receptor (CAR) on their surface [6]. In contrast to the T cell receptor (TCR), CAR enables the recognition of antigens present on cancer cells, independently of major histocompatibility complex (MHC) molecules, thus preventing cancer cells from escaping from the surveillance of the immune system due to the reduced expression of MHC on their surface [7,8,9]. The CAR is composed of four regions, namely: the extracellular antigen-binding domain usually made of a single-chain variable fragment (scFv), the hinge (the spacer region), which increases flexibility and allows the CAR to be properly matched to the target antigen, the transmembrane domain, and the intracellular signaling domain [4,6,8]. The CAR construct was modified so as to increase the efficiency and expansion of CAR-T cells in the immunosuppressive tumor microenvironment (TME) [6,10]. Currently, there are five generations of CARs, differing mainly in the structure of the intracellular signaling domain [8,10]. The comparison of the structure of CAR of different generations is presented in Figure 1. Additionally, the fourth-generation CAR-T cells are engineered to produce the immunostimulatory transgene [10]. This transcription factor brings about inducible or constitutive inflammatory cytokine production (e.g., interleukins 12 (IL-12), IL-18, IL-7, IL-15, or IL-23), following the activation of fourth-generation CAR-T cells [10,11]. For this reason, these cells are also called T cells redirected for universal cytokine-mediated killing (TRUCKs) [10].

The results of clinical trials of the use of CAR-T cells led to the approval by the Food and Drug Administration (FDA) of six drugs based on CAR-T technology for the treatment of patients with relapsed and/or refractory B cell malignancies [11]. This prompted researchers to conduct tests on the use of CAR-T cells in the treatment of other malignancies, including acute myeloid leukemia (AML).

AML is a malignancy of the hematopoietic system of a heterogeneous nature [12]. The disease is caused by mutations resulting in the proliferation of cancer cells derived from progenitor cells of the myeloid lineage [13]. AML is more common among elderly patients, the median age of patients at diagnosis being 68–71 [12,14]. However, 1/3 of AML cases are diagnosed in patients under 50 years of age [14]. AML is also responsible for about 8–10% of cancers in children; the majority of cases concern adolescents and newborns during the first four weeks of life [15,16]. The diagnosis of AML is possible when at least 20% of blasts are found in the bone marrow (BM)/peripheral blood, or when the presence of mutations characteristic of AML, namely, t(8;21), inv(16), t(16;16) or t(15;17) [13,17], is observed. 

AML is associated with a higher risk of resistance for standard treatment or relapse [18]. From 10% to 40% of young patients and from 40% to 60% of patients over 60 years of age do not respond to induction treatment, which is associated with a poor prognosis [18]. Approximately 40% of patients undergoing hematopoietic stem cells transplantation (HSCT) will also develop AML recurrence [18]. The 5-year relative survival rate in AML patients was estimated at 31.7% [19]. The number of long-term survivors in elderly AML patients, over 60 years old, amounts to 10–15% [20]. Due to the insufficient efficacy of standard procedures in the treatment of AML, new targeted therapies are sought, the use of which synergistically with other therapeutic agents might increase the efficacy of AML treatment. The objective of the following review is to present the latest reports on the results of CAR-T cells directed against CD33, CD123, FLT3 and CLL-1 present on leukemic cells in the treatment of AML, the emerging challenges, as well as the prospects for the future.

## 2. The Possibility of Using CAR-T Cells in AML Therapy

CAR-T cells have already been used in clinical trials in patients with relapse AML. One of the first promising results was presented in 2019 by Danylesko et al. [21] An AML patient with t(8;21) (q22;q22.1) after relapse after alloHSCT was given the second-generation CAR-T cells with the cluster of differentiation 28 (CD28) as a co-stimulatory domain in a dose of 1 × 10^6^ CAR T cells/kg. Due to the patient’s aberrant expression of cluster of differentiation 19 (CD19) on AML blasts, a CAR specific for the CD19 antigen was used. On day 3 after the administration of CAR-T cells, the patient developed cytokine release syndrome (CRS) grade 3, controlled with tocilizumab. The patient achieved clinical and molecular remission on day 28 after the administration of CAR-T cells [21]. However, CD19 expression on AML blasts is restricted mainly to patients with t(8;21). Immunophenotyping of one hundred and eighty-eight samples from AML-type M_2_ patients showed CD19 expression in 29.6% of cases, while in another study, five out of seventy-nine AML pediatric samples showed CD19^+^ expression when assessed by flow cytometry [22,23].

The identification of the correct target antigen for CAR-T cells is essential for a successful therapy. The ideal target antigen would be a molecule found abundantly on all subpopulations of cancer cells and absent from or minimally present on healthy tissues. The heterogeneity of AML combined with the propensity of leukemic cells to change the expression of surface antigens with the progression of the disease makes it difficult to identify the target antigen [24]. Table 1 presents selected antigens frequently expressed on leukemic cells in AML. Many of the antigens present on leukemic cells in AML are simultaneously present on healthy cells of the myeloid lineage, which can cause off-target CAR-T cell toxicity, i.e., myelosuppressive effect [8,16]. 

### 2.1. Mechanism of Action of CAR-T Cells

**Figure 2:**
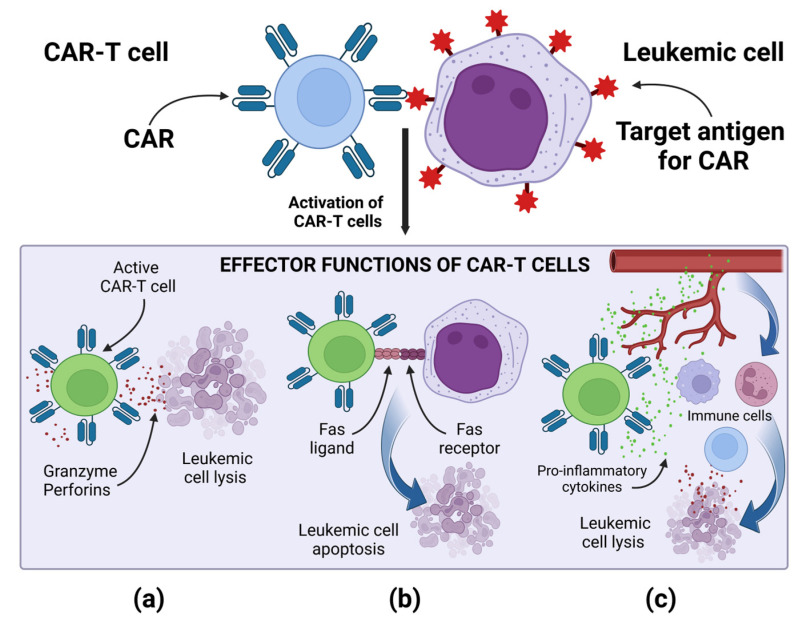
Effector mechanism of CAR-T cells: (**a**) Release of perforins and granzymes by active CAR-T cells leads to leukemic cell lysis. (**b**) Active CAR-T cells via the Fas and FasL pathway lead to leukemic cell apoptosis. (**c**) Active CAR-T cells secrete pro-inflammatory cytokines, enhancing the anti-cancer response of other cells of the immune system. Image created with biorender.com (accessed on 22 April 2023). CAR—chimeric antigen receptor, CAR-T—T cell with chimeric antigen receptors.

Regardless of the type of the target antigen, the mechanism of action of CAR-T cells is similar, and it is based on the specific binding of the CAR to the target antigen present on the tumor cell. Then, a non-classical immune synapse is formed [37]. The purpose of co-stimulatory domains is to mimic co-stimulatory signals secreted by antigen presenting cells (APCs) in the TCR-mediated antigen presentation process, during which receptors present on T lymphocytes bind to their ligands present on APCs [37,38]. Signal transduction leading to CAR-T cell activation is enabled by the CD3ζ cytoplasmic domain and the co-stimulatory signaling domain. Depending on the co-stimulatory domain used, different co-stimulatory signals are achieved [38]. Signaling through the CD28 co-stimulatory domain is mediated by phosphoinositide 3-kinase, while signaling through the tumor necrosis factor receptor superfamily 9 (4-1BB) co-stimulatory domain is mediated by tumor necrosis factor receptor-associated factors and nuclear factor-κB (NF-κB) [37,38]. The CD3ζ cytoplasmic domain contains three immunoreceptor tyrosine-based activation motifs (ITAMs). After the CAR binds the antigen, ITAMs is phosphorylated by the lymphocyte-specific protein tyrosine kinase (Lck). The phosphorylated ITAMs is joined by the ζ-associated protein of 70 kDa (ZAP70), which is also phosphorylated by Lck, causing its activation [39]. Active ZAP70 mediates downstream signaling, which ultimately leads to the activation of effector functions of CAR-T cells [37,38,39]. The main effector mechanism causing the lysis of cancer cells is the secretion of perforins and granzymes by CAR-T cells [37]. CAR-T cells, when activated, can also induce apoptosis of cancer cells negative for the target antigen via the Fas and FasL pathway, thanks to which CAR-T cells can perform effector functions in a cancer tumor, showing the heterogeneous expression of antigens [37]. After activation, CAR-T cells secrete pro-inflammatory cytokines, thus increasing the anti-cancer response of other cells of the immune system. A special role in cytokine-mediated killing is played by CAR-T cells of the fourth generation, which additionally have a transcription factor that brings about inducible or constitutive inflammatory cytokine production [10,37]. For example, the release of IL-12 by TRUCKs increases the cytotoxic efficiency of T lymphocytes, by inducing the production of interferon γ (IFN-γ), perforins and granzymes, and inhibits the proliferation of regulatory T lymphocytes (Treg), which are part of TME. TRUCKs can also secrete IL-15, which increases the expansion and survival of T lymphocytes, and IL-18, which, by modulating TME cells, can sensitize cancer cells to effector cells of the immune system [40]. The effector mechanisms of CAR-T cells are presented in Figure 2.

### 2.2. CAR-T Cells Anti-CD33

CD33 (Siglec-3) belongs to the sialic acid-binding Ig-like lectin family [26]. It is a glycoprotein consisting of two extracellular domains, V-set Ig-like (IgV) and C2-set Ig-like (IgC2), a transmembrane domain and an intracellular domain. In some patients, CD33 is expressed as a shorter isoform, which is lacking in IgV (CD33-D2) [25]. CD33 is normally present on myeloid progenitor cells [41]. It is also found on cancer cells in AML in more than 90% of cases [25]. Particularly relevant for relapsed/refractory (R/R) AML therapy is the fact that this antigen is also expressed on leukemic stem cells (LSCs) [26]. CD33 has already been used as an antigen in targeted therapy using a conjugated antibody—gemtuzumab ozogamicin (GO), which proved to be safe and effective in several clinical trials, leading to its approval for the treatment of AML by the FDA [25,42]. Therefore, CD33 seems to be a suitable target for the treatment of AML, including the use of CAR technology. However, it is worth bearing in mind that the expression of CD33 on BM progenitor cells may be associated with off-target side effects, and addressing this issue is one of the major challenges in anti-CD33 CAR-T cell therapy.

**Preclinical studies.** O’Hear et al. created the second-generation anti-CD33 CAR-T cells with the 4-1BB co-stimulatory domain, and tested their ability to kill leukemic cells in vitro and in vivo. In the presence of tumor cell lines and primary tumor cells, the constructed CAR was highly effective, eliminating these cells with an effector to target (E:T) ratio < 1, even as low as 1 effector cell for 20 targets. The sensitivity of CAR-T cells was also high, as cytolysis was observed regardless of the level of CD33 expression. In a mouse model of AML, anti-CD33 CAR-T cells prevented tumor growth, leading to significantly prolonged survival, although each mouse eventually developed a tumor. The authors showed that CAR-T cells also targeted normal CD33^+^ BM cells, demonstrating that these CAR-T cells may have a myelosuppressive effect [43]. In another study, Kenderian et al. obtained similar results showing the high efficiency of lentiviral transduced anti-CD33 CAR-T in vitro and in vivo. The authors emphasized the significant myelotoxicity of these cells in mouse xenografts. In order to minimize this side effect, the authors developed the transiently expressed RNA-modified CAR-T anti-CD33. The anti-tumor activity of these cells was similar compared to lentivirally transduced CAR-Ts. However, the concentration of T cells gradually decreased over seven days, which was associated with a decrease in cytotoxicity. Next, the combination of RNA-modified CAR-T with lymphodepleting chemotherapy (cyclophosphamide—60 mg/kg intraperitoneally) was tested. Interestingly, this approach resulted in better responses and a prolonged survival [41]. 

Some researchers have compared the effectiveness of CARs composed of different co-stimulatory domains or different binding domains. Li et al. created three anti-CD33 CAR-Ts: two second-generation CARs with a CD28 or 4-1BB co-stimulatory domain, and one third-generation CAR with both domains. All constructs showed good proliferation and anti-tumor activity against AML cell lines, killing leukemic cells with an E:T ratio as low as 1:8. However, the authors observed some differences between individual CARs with different co-stimulators. Constructs containing 4-1BB had an increased central memory compartment. Interestingly, the CD28 CAR-T cells in ex vivo conditions were predisposed to exhaustion, while the 4-1BB CAR-T cells appeared to be resistant to it. These results could suggest that CAR-T cells combined with the 4-1BB co-stimulatory domain may perform better than when they are combined with other domains [44]. In another study, by Qin et al., the authors constructed six different CAR-T cells from one of the three scFvs from clinically tested antibodies—GO, lintuzumab and M195—with either CD28 or 4-1BB as co-stimulatory domains. The production of cytokines and the cytotoxicity of CAR-T cells incubated with human CD33^+^ AML cell lines were observed, but no one of the six tested constructs was superior. In in vivo studies, M195-based CAR-T cells showed a lower level of efficacy in tumor eradication; therefore, further research on this construct was abandoned. GO-CD33 CAR-T cells showed significant toxicity in animal models, while lintuzumab-CD33 CAR-T cells were well tolerated and showed great anti-leukemia activity. In addition, a higher inhibition of leukemia proliferation was observed using the CD28 co-stimulatory domain compared to 4-1BB. Consequently, the authors selected lintuzumab-CD28/CD3ζ anti-CD33 CAR-T cells for the early phase clinical trial in children and adolescents/young adults with R/R AML [45]. 

**Figure 3:**
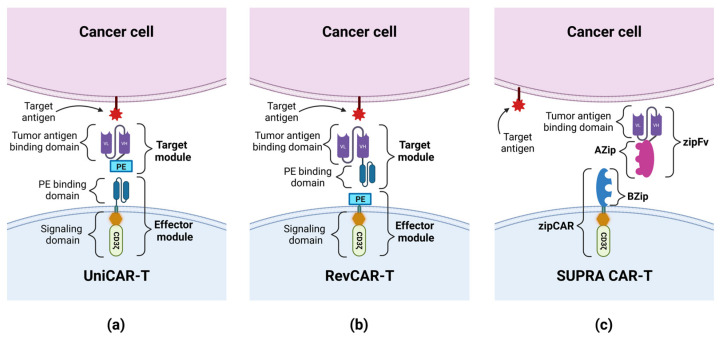
The construction of UniCAR, RevCAR and SUPRA CAR: (**a**) UniCAR consists of a universal effector module (EM) and a tumor-specific target module (TM). EM is composed of a signaling domain and a peptide epitope-binding domain. TM is composed of a peptide epitope (PE) and a tumor antigen-binding domain. The administration of TMs targets the UniCAR cell to the tumor cell, and enables the activation of cytotoxic mechanisms. (**b**) RevCAR consists of a universal EM and a tumor-specific target module (RevTM). EM is composed of a signaling domain and a peptide epitope (PE). TM is a bispecific target module composed of 2 scFvs: a peptide epitope-binding domain and a tumor antigen-binding domain. The administration of RevTM targets the RevCAR cell to the tumor cell, and enables the activation of cytotoxic mechanisms. (**c**) SUPRA CAR consists of a universal receptor with a leucine zipper adapter (zipCAR), and scFv with a leucine zipper adapter (zipFv) molecule, directed against the target antigen. A leucine zipper (AZip), linked to scFv, can link to the cognate a leucine zipper (BZip) present on the zipCAR. The zipFv binding to the target antigen and dimerizing with the zipCAR results in the activation of the SUPRA CAR cell. The administration of the zipFv targets the SUPRA CAR cell to the tumor cell, and enables the activation of cytotoxic mechanisms.

As was mentioned earlier, the presence of CD33 on normal cells may result in side effects of the CAR-T therapy. One way to reduce toxic effects is to use the Universal CARs (UniCARs). UniCAR consists of two parts: a universal effector module (EM) and a tumor-specific target module (TM). EM is composed of intracellular signaling domains and an extracellular peptide epitope binding domain, which is physiologically absent on the cell surface, so UniCAR cells are devoid of cytotoxic activity until the administration of TM composed of a peptide epitope and a tumor specific antigen-binding domain. The administration of TMs targets the UniCAR cell to the tumor cell, and enables the activation of cytotoxic mechanisms (see Figure 3) [46,47]. Cartellieri et al. created the UniCAR platform targeting CD123 and CD33. The advantage of this method is its ability to select which antigen CAR-T cells are to be targeted at any given time by using a specific TM. In addition, this method gives the option to disable CAR-T cells’ activity by withdrawing TMs supply, which may give the necessary time to restore normal hematopoiesis, damaged by CAR-T cells [48]. Another solution to this problem was proposed by Kim et al., who hypothesized that the CD33 knockdown (KO) from normal hematopoietic stem and progenitor cells (HSPCs) would produce a hematopoietic system resistant to anti-CD33 CAR-T cells therapy. After the implantation of CD33-defficient HSPCs into immunodeficient mice, normal cell differentiation was demonstrated, and normal myeloid function was observed after autologous CD33 KO HSPC transplantation in rhesus macaques. The activity of anti-CD33 cells was then tested and showed a high efficiency in terms of the elimination of leukemic cells, but no targeting of CD33-defficient cells was observed; therefore, no signs of myelotoxicity were noticed [49]. In another study, Liu et al. investigated the use of CD33 KO by analyzing the activity of the third-generation anti-CD33 CAR-T cells and GO. Compared to the second-generation CARs, the tested cells exhibited higher viability, increased proliferation, and stronger cytotoxicity. Importantly, both the third-generation CAR-T cells and GO attacked CD33^+^ AML cells while sparing CD33-defficient cells [25].

**Clinical studies.** The first report on the outcome of anti-CD33 CAR-T treatment in a patient with AML dates back to 2015, and was presented by Wang et al. A patient with chronic pancytopenia, ineligible for chemotherapy, was included in this clinical trial. The authors constructed the second-generation anti-CD33 CAR-T cells with the 4-1BB co-stimulatory domain. Without any conditioning therapy, he was given CAR-T cells in a total dose of 1.12 × 10^9^ cells on four consecutive days. After each cell infusion, the patient experienced chills and a high fever, reaching 42 °C. The fever recurred on day 9 after the first infusion, but the patient’s condition stabilized after the administration of etanercept. Pancytopenia was also exacerbated after CAR-T cell administration and the patient required a blood transfusion. CAR-T therapy reduced the blast rate from >50% to <6% after two weeks. Later, however, the number of blasts increased—22% at week 3, 27% at week 5, and 70% at week 9. The patient progressed and died 13 weeks after anti-CD33 CAR-T infusion [50]. Although the treatment was unsuccessful, the initial cytotoxicity of CAR-T against leukemic cells, as well as the lack of life-threatening complications, could encourage further research on anti-CD33 CAR-T therapy in AML. 

In another phase I clinical trial, the feasibility and safety of anti-CD33 CAR-T cells with 4-1BB co-stimulatory domain in patients with R/R AML was investigated by Tambaro et al. Ten patients were included in the study; eight of them underwent aphaeresis. In the case of the remaining two patients aphaeresis was impossible due to the rapid progression of the disease. In the case of patients with aphaeresis, four of them produced anti-CD33 CAR-T cells that met the criteria for infusion. Finally, three patients received anti-CD33 CAR-T cells, because the fourth patient died before receiving the cells. Two patients developed CRS and one developed immune effector cell-associated neurotoxicity (ICANS). Other adverse events associated with anti-CD33 CAR-T cells included grade 3 tumor lysis syndrome, grade 2 mucositis, and grade 1 tachycardia in one patient; the second patient developed grade 2 intermittent orthostatic hypotension, grade 2 increased bilirubin and grade 3 increased alanine aminotransferase (ALT), and aspartate aminotransferase (AST). Interestingly, the last patient experienced no toxicity. Despite the detection of anti-CD33 CAR-T cells in the blood, none of the patients met the treatment response criteria. All three patients died due to disease progression [26]. 

Another clinical study analyzed the PRGN-3006 UltraCAR-T drug. It is characterized by the expression of anti-CD33 CAR, membrane-bound Il-15 and kill switch. This clinical trial included fifteen adult patients with R/R AML and some higher-risk myelodysplatic syndromes. The patients were divided into two cohorts: the first without lymphodepletion, and the second after lymphodepletion. Seven patients developed CRS, including one grade 3 that resolved after the administration of tocilizumab and dexamethasone. One case of grade 2 graft-versus-host disease (GvHD) was observed, which resolved completely after treatment with corticosteroids. Comparing both cohorts, a greater expansion of PRGN-3006 was noted in the second one. No objective responses were observed in the first cohort, while in the second one the objective response rate (ORR) was 50%. Currently, recruitment for the further study of PRGN-3006 is ongoing, thanks to which the data on safety and efficacy will be updated [51]. 

### 2.3. CAR-T Cells Anti-CD123

CD123 is the alpha chain of the interleukin 3 receptor (IL-3Rα) [52]. The extracellular part of the IL-3R alpha subunit consists of three domains, two of which bind interleukin 3, while the N-terminal domain prevents the spontaneous dimerization of IL-3Rα with the major signaling component, βc subunit [53,54]. The results of the studies evaluating the expression of CD123 on hematopoietic stem cells (HSCs) were contradictory; however, it is assumed that CD123 is present to a low degree on some subsets of HSCs [32,54]. Erythroid progenitors most often do not express this antigen, while it is usually observable on granulomonocytic progenitors [32]. IL-3Rα has also been reported on the surface of plasmacytoid dendritic cells, basophils, monocytes, eosinophils and myeloid dendritic cells. In addition, CD123 is found on some endothelial cells [55]. The antigen is a potential target for the immunotherapy of AML because it shows high levels of expression on AML blasts and LSCs, often associated with FLT3-internal tandem duplication (ITD) or nucleophosmin 1 mutations [54,56]. The overexpression of CD123 on blasts translates into a poor prognosis due to possible resistance to chemotherapy [54]. Despite significant differences in antigen expression between AML blasts and normal cells, anti-CD123 CAR-T cells can cause endothelial damage and have an inhibitory effect on normal hematopoiesis [57,58].

**Preclinical studies.** Gill et al. in 2014 presented the results of one of the first studies confirming the effectiveness of anti-CD123 CAR-T cells with the 4-1BB co-stimulatory domain in vitro and in vivo on a mouse AML xenograft model. The administration of a single dose of anti-CD123 CAR-T cells to animals increased their survival compared to the control group, and resulted in the elimination of leukemic cells, including initially CD123^dim^ AML populations, which may be due to the upregulation of CD123 on blasts as the disease progressed. The study also showed that anti-CD123 CAR-T cells led to the eradication of normal hematopoiesis in a xenograft model [59]. In order to increase the safety of anti-CD123 CAR-T cells and simultaneously avoid their toxic effects beyond target, Tasian et al. proposed CAR-T cell depletion after the eradication of leukemic cells. It can be achieved in three different ways: (i) the application of transiently active anti-CD123 matrix RNA electroporated CAR-T cells (RNA-CART123); (ii) CAR-T cells clearance after therapy with alemtuzumab; (iii) CAR-T cells clearance after therapy with rituximab in the case of the co-expression of CD20 on CAR-T cells (CART123-CD20). The studies carried out by the authors confirmed that all proposed CAR-T cells effectively eradicate AML in mouse xenograft models, and the depletion of CAR-T cells did not lead to leukemia recurrence. It has also been shown that the ablation of CAR-T cells enables the renewal of HSCs in normal human hematopoiesis xenograft models [60].

Some researchers suggested that the use of the DNA methyltransferase inhibitors synergistically with CAR-T cells might improve their efficiency. You et al. in their studies have shown that decitabine (DAC) promotes CD123 CAR-T differentiation into superior naive and memory phenotypes, and inhibits the expression of DNA methyltransferase 3a, which takes part in T cell exhaustion [61]. In their study, Khawanky et al. used another DNA methyltransferase inhibitor, azacitidine (AZA). The authors constructed the third-generation anti-CD123 CAR-T cells with CD28 and tumor necrosis factor receptor superfamily member 4 (OX40) as co-stimulatory domains. The study showed that the use of AZA increased the expression of CD123 on leukemic cells and decreased the expression of the immune checkpoint, cytotoxic T cell antigen 4 (CTLA-4), on CAR-T cells. Mice receiving this treatment regimen showed improved AML elimination in vivo and long-term control of AML xenograft models [62]. 

One of the problems, limiting the effectiveness of CAR-T cells, is the escape of leukemic cells from CAR-T cells surveillance through the loss of the expression of the target antigen for CARs. The prevention of this may be achieved by using Dual-CAR cells that co-express two independent types of CARs specific for different antigens or a bispecific tandem CAR (TanCAR-T cell), which has one type of CAR with two binding domains that target two different antigens [63,64]. In their work, Petrov et al. presented the anti-leukemic activity of anti-CD123-CD33 Dual-CAR-T cells in vivo, which cause the elimination of leukemia blasts and LSCs in leukemia cell lines with a nearly 100% killing rate. The administration of these cells to mice xenograft models of leukemia improved their survival when compared to the control group. In order to check the safety profile of the therapy and prevent myeloablation, the authors used the alemtuzumab-mediated depletion strategy, thanks to which 90% of anti-CD123-CD33 Dual-CAR-T cells were eliminated six hours after the administration of the alemtuzumab to mice [65]. In turn, Ghamari et al. constructed TanCAR-T cells targeting CD123 and the scFv-binding folate receptor beta (FRβ). The study showed that the co-culturing of TanCAR FRβ-CD123 T cells with AML blasts resulted in the increased secretion of IFN-γ and interleukin 2 (IL-2) compared to anti-CD123 CAR-T cells [66].

Preclinical studies have also been conducted to investigate the feasibility and efficacy of UniCAR-T anti-CD123 drugs. Loff et al., in an in vivo study, showed that UniCAR-T cells, compared to CAR-T cells, have a lower hematotoxic effect against HSCs, while maintaining effectiveness against AML cells, due to the limited administration of TMs. Using UniCAR-T with CD28 as a co-stimulatory domain in combination with a CD123-specific TM (TM123) in an AML cell line (MOLM-13) xenograft mouse model, the authors observed a decrease in the number of AML cells in the BM and an increase in survival in the group of mice treated with TM123 compared to the control group (median survival 74 days vs. 53 days). However, UniCAR-T cells failed to achieve a complete remission (CR) of the disease, and the mice treated with TM123 died of AML later, despite confirming the long-term efficacy of UniCAR-T cells in an ex vivo test [56]. Therefore, work began on optimizing the structure of TMs targeting CD123, which could increase the effectiveness of UniCAR-T. Meyer et al. constructed a CD123-specific TM variant additionally containing the trimeric single-chain 4-1BBL (TM123-4-1BBL), which, apart from directing UniCAR-T to CD123^+^ cells, would co-stimulate them by binding to the 4-1BB receptor on T cells, improving their in vivo stability. Targeting UniCAR-T cells by TM123-4-1BBL compared to targeting by TM123 improved the efficiency and cytotoxic functions of UniCAR-T in an in vitro assay simulating the conditions of T cell depletion. During the next stage of research, conducted on a mouse MOLM-13 xenograft model, it was shown that the use of TM123-4-1BBL increases the functionality of UniCAR-T. The median survival of the untreated control group was 21 days, while the median survival of the two UniCAR-T groups with TM123 or TM123-4-1BBL was the same—31 days. Ultimately, all animals treated with UniCAR-T showed disease progression. It is worth noting, however, that the phenotype of most UniCAR-T cells in the TM123-4-1BBL group was shifted towards CD8^+^ effector lymphocytes, while the phenotype of cells in the TM123 group was mainly CD4^+^ helper lymphocytes [67]. The modification of UniCAR designed to reduce the size of the gene encoding the CAR construct introduced into T lymphocytes is the Reversed CAR (RevCAR). RevCAR, like UniCAR, consists of two parts: EM and TM. The difference between UniCAR and RevCAR is the location of the peptide epitope and peptide epitope-binding domain. In RevCAR, the peptide epitope is an EM element, while the peptide epitope-binding domain is one of the TM elements, whereas in UniCAR it is the other way round (see Figure 3) [68]. Kittel-Boselli et al., in their work, presented the results of research conducted on RevCAR-T cells. By administering two different TMs (one RevTM-CD123 and the other RevTM-CD33), the same RevCAR-T cell could be targeted by the TM to a CD123^+^ or CD33^+^ tumor cell and, by activating the effector mechanism, lead to its elimination. The effectiveness of RevCAR-T cells was confirmed in in vitro and in vivo tests. In order to reduce the off-target effect, the authors constructed Dual-RevCAR cells expressing two different RevCARs: SIG (activating signaling) and COS (co-stimulatory signaling), which made AND logic gate targeting possible. SIG RevCAR-E7B6-3z pairs with RevTM CD123-7B6 and COS RevCAR-E5B9-28 with RevTM CD33-5B9. The stimulation of SIG RevCAR alone, with a low density of the CD123^+^ antigen on the target cells, is insufficient to activate the effector mechanisms of Dual-RevCAR T cells. Under such conditions, for the complete activation of Dual-RevCAR T cells, an activation and co-stimulation signal must be transmitted, thus the effector mechanisms of Dual-RevCAR T cells will occur against leukemic cells co-expressing CD123 and CD33, which was confirmed in in vitro tests [69].

Sugita et al. in their work constructed gene-edited CAR-T cells anti-CD123 (UCART123), which do not express TCRαβ, in this way reducing the risk of GvHD in the recipient after the administration of allogeneic cells. Researchers confirmed the effectiveness and specificity of UCART123 cells in eliminating CD123^+^ leukemic cells. The co-culturing of UCART123 cells with AML cells lysed over 70% of the tumor cells (E:T ratio 0.5:1) after 24 h. For off-target cytotoxicity assessment, UCART123 cells were also co-cultured with cord blood (CB) samples from healthy donors, resulting in the lysing of 9.2% of CB cells (E:T ratio 0.5:1) after 24 h. The cell activity of UCART123 in PDX-AML2 and PDX-AML37 mice models (1 million UCART123 or 2.5 million dosage) was also assessed. The administration of UCART123 cells significantly improved the overall survival (OS) of all study groups compared to control groups. At the end of the experiment, the mice that received a higher dose of UCART123 were disease-free, while some of the mice treated with 1 million cells were relapsing, which was probably due to the lack of UCART123 persistence in the body. To evaluate the selectivity of UCART123 cells in vivo, a mouse xenograft model containing both normal and leukemic cells was created. The study group was then given 1 million UCART123 cells. The evaluation of the mouse BM showed that the majority of leukemic cells were eliminated, but most of the normal BM cells were retained (on average, close to a 2-fold decrease in CD33^+^ cells and completely preserved lymphoid lineages) [55].

**Clinical studies:** In 2019, Sun et al. decided to use the donor-derived second-generation anti-CD123 CAR-T cells combined with chemotherapy as the conditioning regimen for haploidentical HSCT in a patient with t(16;21) (p11;q22) AML relapse after allo-HSCT and a total of nine prior lines of ineffective therapy. The donor of T lymphocytes, later modified to express CAR, was the patient’s father (5/10 human leukocyte antigen (HLA) loci matching and ABO incompatibility with the patient). The patient received CAR-T cells with 4-1BB as a co-stimulatory domain at a total dose of 1.1 × 10^8^ cells (80.2% CAR^+^). Twenty-four hours after infusion, the patient developed grade 3 CRS, treated with tocilizumab, but after four days the patient developed CRS grade 4, controlled by the infusion of methylprednisolone and anti-thymocyte globulin. The BM blast count decreased from 40.8% to 10.3% six days after the anti-CD123 CAR-T cells infusion. On day 6, the patient received granulocyte colony-stimulating factor-mobilized peripheral blood stem cells (G-PBSC), also isolated from the father. Due to poor engraftment, the infusion of G-PBSC was repeated on day 24. The patient achieved complete remission with incomplete blood count recovery (CRi), full donor chimerism, and myeloid engraftment. At 38 days after the infusion of CAR-T cells, the patient began to have the symptoms of acute GvHD and, despite treatment, died on day 62 [33]. In January 2020, clinical trials NCT04230265 began, in which patients with R/R AML with at least 20% CD123^+^ blasts in the BM received the autologous UniCAR02-T cells and TM123. The treatment regimen consists of the daily administration of TM123 for 25 days and a single administration of UniCAR02-T cells one day after the start of the TM123 infusion. So far, eight patients have been tested. Two of them were excluded from the study due to a lack of CD123^+^ expression. Of the remaining six eligible patients, two patients died before receiving treatment. To date, treatment has been completed in three patients. Patient 1, aged 54, with 26% blasts in BM (80% CD123^+^), received a single dose of 100 × 10^6^ UniCAR-T cells and 0.5 mg TM123 every day for 25 days. After the administration of the preparation, the patient achieved partial remission (13% blasts in BM). Patient 2, aged 65, with 20% blasts in BM (26% CD123^+^), received a single dose of 250 × 10^6^ UniCAR-T cells and 0.5 mg TM123 every day for 25 days. After the administration of the preparation, the patient achieved CRi (0% blasts in BM). Patient 3, aged 80, with 30% blasts in BM (80% CD123^+^), also received a single dose of 250 × 10^6^ UniCAR-T cells, but the dose of TM123 was increased to 1 mg per day for 25 days. After the administration of the preparation, similarly to Patient 2, he achieved CRi (2% blasts in BM). This patient relapsed after one month, and due to persistent UniCAR02-T Cells in his system, the patient received a second cycle of TM123. No symptoms of neurotoxicity occurred in any of the three patients, while Patient 2 and Patient 3 developed CRS grade 1 after the administration of the preparation. Patient 4 is currently being treated with the use of a single dose of 500 × 10^6^ UniCAR02-T Cells and 1 mg TM123 every day for 25 days [70]. 

### 2.4. CAR-T Cells Anti-FLT3

**Figure 4:**
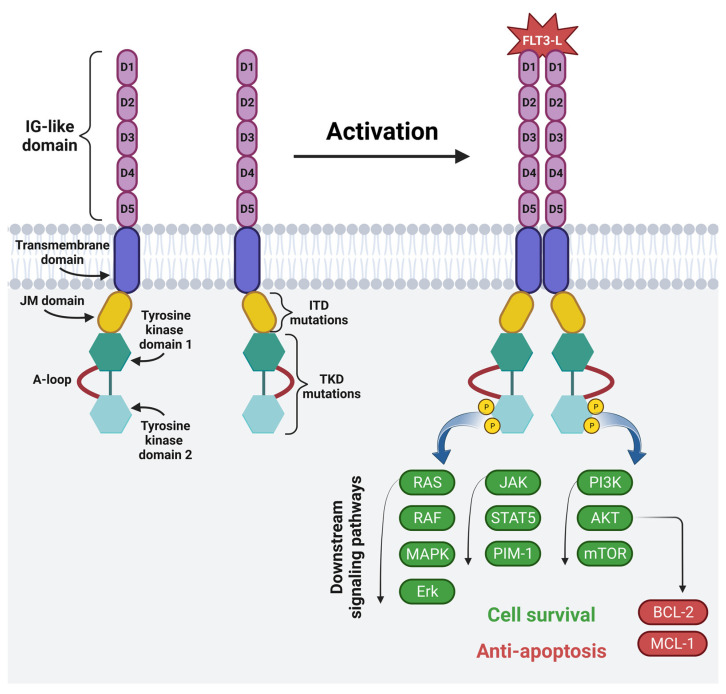
The structure and mechanism of activation of fms-like tyrosine kinase 3: FLT3 consists of five IG-like domains (extracellular part), the transmembrane domain and intracellular part, which includes the juxtamembrane domain (JM domain), two tyrosine kinase domains (TKD1 and TKD2) and an activating loop. The most common FLT3 mutations are internal tandem duplication in the JM domain and point mutations or deletion in the TKD. Physiologically, FLT3 becomes activated upon binding to the FLT3 ligand. Ligand binding causes the homodimerization of the receptor. This is followed by phosphorylation and the activation of RAS/RAF/MAPK/Erk, JAK/STAT5/PIM-1 and PI3K/AKT/mTOR intracellular signaling pathways, which regulate cell proliferation, differentiation and apoptosis. Image created with biorender.com (accessed on 22 April 2023). D1—domain one, D2—domain two, D3—domain three, D4—domain four, D5—domain five, JM domain—juxtamembrane domain, A-loop—an activating loop, ITD—internal tandem duplication, TKD—tyrosine kinase domain, FLT3L—FMS-like tyrosine kinase 3 ligand, P—phosphorus, RAS—from “Rat sarcoma virus”, RAF—rapidly accelerated fibrosarcoma kinases, MAPK—mitogen-activated protein kinase, Erk—extracellular-signal-regulated kinase, JAK—janus kinase, STAT5—signal transducer and activator of transcription 5, PIM-1—proto-oncogene serine/threonine-protein kinase, PI3K—phosphoinositide 3-kinase, AKT—protein kinase B, mTOR—mammalian target of rapamycin, BCL-2—B-cell lymphoma 2, MCL-1—induced myeloid leukemia cell differentiation protein.

The FMS-like tyrosine kinase (FLT3) is a membrane-bound glycosylated protein and a member of the class III receptor tyrosine kinase family [71,72]. FLT3 is involved in normal hematopoiesis by controlling cell survival, proliferation and differentiation [71,72,73]. It is mainly expressed on HSCs and myeloid cells, but it is also expressed on blasts in 54–92% of AML patients [24,34,71,73]. The mutations of the *FLT3* gene located on chromosome 13 are often present in AML patients, and among them ITD occurs in 15–30% of patients, and a mutation in the tyrosine kinase domain (TKD) occurs in 5–10% of patients. These mutations lead to the continuous activation of the FLT3 receptor, leading to the increased survival and proliferation of leukemic cells [73]. This, in turn, is associated with a poor prognosis and a high risk of leukemia recurrence, especially in the case of ITD, for which the five-year event-free survival is 12% and the OS amounts to 16.6% [74]. The structure and mechanism of the activation of the FLT3 receptor is shown in Figure 4.

**Preclinical studies.** One of the first studies on the evaluation of anti-FLT3 CAR-T in vitro and in vivo was published by Chen et al. The authors created the second-generation CAR, containing CD28 as a co-stimulatory domain and variable regions of heavy and light chains derived from a hybridoma. The cytotoxicity of the resulting cells was assessed in the presence of six different FLT3-expressing cell lines and in the presence of FLT3^+^ primary AML blasts. In both cases, cytotoxicity was significant because CAR-T cells effectively eliminated FLT3^+^ targets, accompanied by an increased production of IFN-γ. In in vivo studies, the administration of manufactured CAR-T cells to mice that had been previously injected with MOLM-13 cells resulted in significant anti-leukemic activity and prolonged survival, with a survival rate of 100% on day 80. Similar results were achieved in mice with FLT3^+^ AML patient blasts, and the survival rate amounted to 100% on day 120, while all control group mice died before day 90 [75]. Similar outcomes were obtained in the work of Niswander et al. They created anti-FLT3 CAR-T cells and confirmed their high efficiency in eliminating AML cell lines, as well as the eradication of leukemia in MV4-11 and MOLM-14 AML xenograft models [76].

Some authors decided to target FLT3 by constructing CAR-T cells based on the natural ligand of this antigen. Wang et al. created the second-generation CAR-T cells with a 4-1BB co-stimulatory domain, and chose the FLT3 ligand (FLT3L)-binding domain as the antigen-binding domain. By culturing FLT3L CAR-T cells with different AML FLT3^+^ cell lines, differences in their cytotoxicity and leukemic cell survival were observed. It turned out that CAR-T cells showed significant cytotoxicity and effectively killed leukemic cells with the FLT3-ITD mutation (MV4-11 and MOLM-13 lines), while wild-type FLT3 (WT-FLT3) leukemic cells (REH and THP-1 lines) were able to survive even at the highest E:T ratio of 1:1. The authors explained that FLT3L activated the FLT3 downstream signaling pathway in WT-FLT3 cells, which was confirmed by increased Erk (extracellular-signal-regulated kinase) phosporylation in these cells when cultured with FLT3L CAR-T cells. The activation of this pathway led to increased proliferation and cell survival. In contrast, this phenomenon was not observed for cells with FLT3-ITD, where the activation of the FLT3 pathway is ligand-independent. Based on these results, it can be concluded that FLT3L CAR-T cells may be an effective treatment for AML with the FLT3-ITD mutation [77]. Another research group that decided to use FLT3L as a binding domain constructed the third-generation CAR-T for this purpose, with 4-1BB and inducible T cell co-stimulator as the co-stimulatory domains. In contrast, full-length human FLT3L was used as the binding domain. The generated CAR-T cells showed specific cytotoxicity to the FLT3^+^ cell line (THP-1), but not to the FLT3^−^ line (U937). To confirm the aforementioned observations, soluble human FLT3L was added to the co-culture of FLT3L CAR-Ts and THP-1 cell lines. The soluble FLT3L led to the inhibition of CAR-T cells, while a reduction in its concentration led to the disappearance of this effect, which proved the specificity of the created CAR-T cells for FLT3^+^ cells [78].

One way to increase the effectiveness of anti-FLT3 CAR-T cell therapy is to combine it with other treatment methods. Jetani et al. decided to investigate the combination of anti-FLT3 CAR-T cell therapy with the FLT3 inhibitor—crenolanib. FLT3 inhibitor leads to a compensatory increase in the expression of FLT3 on these cells, so the authors hypothesized that the use of crenolanib will increase the efficiency of CAR-T cells. Initially, it was found that when MOLM-13 cells were cultured in the presence of crenolanib, FLT3 expression on the surface of these cells was increased. On the other hand, the removal of the FLT3 inhibitor again reduced FLT3 expression on these cells. In vitro studies showed a higher efficacy of anti-FLT3 CAR-T cells against leukemic cells previously co-incubated with crenolanib. In turn, in in vivo studies, response rate and OS were higher in the group receiving synergistic therapy, compared to anti-FLT3 CAR-T cells alone [79]. Another group of researchers also decided to investigate the combination of CAR-T cells therapy with an FLT3 inhibitor. For this purpose, Li et al. created the second-generation Dual-FLT3scFv/NKG2D-CAR-T (NKG2D—natural killer group 2 member D protein) cells with 4-1BB as co-stimulatory domain, and used gilteritinib—the second-generation FLT3 inhibitor. The generated CAR-T cells targeted FLT3 and natural killer group 2 member D protein ligands NKG2DLs, which are also found on AML cells. As a result, they caused cell death in various AML cell lines, but were especially effective in killing AML cells with the *FLT3* mutation. The efficiency of CAR-T cells was further enhanced when gilteritinib was added. In an AML xenograft mouse model study, the treatment of mice with the synergistic therapy resulted in a higher median mouse survival (35 days), compared to mice treated with CAR-T cells alone, gilteritinib alone, and controls (24, 19 and 15 days, respectively). The authors attributed the increased effectiveness of therapy after the inclusion of gilteritinib to the fact that gilteritinib not only increases the expression of FLT3 on AML cells, but it also, through its interaction with the noncanonical NF-κB2/Rel B signaling pathway, leads to an increase in the expression of NKG2DL on AML cells [74]. Both research groups assessed the effects of these cells on HSCs. Jetani et al., in an in vitro study, observed that anti-FLT3 CAR-T cells lysed ~80% of normal HSCs within 24 h. The authors implanted normal HSCs into NSG-3GS mice and administered anti-FLT3 CAR-T cells after 8 weeks. It was noted that normal HSCs and progenitor cells were removed from the BM [79]. Li et al. incubated HSCs obtained from neonatal CB with CAR-T cells, and observed the lysis of HSCs at 7%, 20% and 23% for E:T ratios of 1:1, 10:1 and 20:1, respectively [74]. These results are contradictory to those published by Wang et al. and Chen et al., who showed that anti-FLT3 CAR-T cells had no negative effect on HSCs and did not affect colony formation [75,77].

In order to reduce the risk of adverse events of anti-FLT3 CAR-T cell therapy, Sommer et al. decided to include a safety switch in the CAR construct. The authors confirmed that the co-culture of anti-FLT3 CAR-T cells led to a significant reduction in the number of HSCs. To increase the safety of this therapy, two mimotopes of rituximab (R2 off-switch) were added to the CAR construct between the hinge region and the scFv. The obtained cells (anti-FLT3 CAR-R2 T) showed no reduced efficiency compared to CAR-T cells without an off-switch. Further studies confirmed the effectiveness of anti-FLT3 CAR-R2 T cells in the eradication of leukemia, and the administration of rituximab resulted in the depletion of CAR-T cells, thus limiting hematotoxicity and enabling BM recovery [80].

### 2.5. CAR-T Cells Anti-CLL-1

C-type lectin-like molecule-1 (CLL-1) belongs to group V of the C-type lectin-like receptor family [81]. It is a type II transmembrane glycoprotein, which is expressed on myeloid lineage cells. Therefore, CLL-1 is also found on AML blasts, and its expression is estimated at 85–92% of AML of M0-M6 French–American–British classes [35]. What is important too is that its expression is also found on LSCs, which are seen as a major cause of treatment failure and AML recurrence [36]. In one study CLL-1^+^CD34^+^C38D^−^ cells were isolated and implanted in non-obese diabetic/severe combined immunodeficiency mice. It turned out that these mice developed leukemia, suggesting that the implanted cells possess LSCs properties [82]. It is also worth mentioning that HSCs do not express CLL-1, although some authors report that a small fraction of these cells may have this antigen on their surface. CLL-1 expression is also not found on lymphoid progenitor cells. However, this is found to a high degree on myeloid progenitor cells and on mature granulocytes and myelocytes [36,81]. Therefore, anti-CLL-1 CAR-T may be an effective therapy targeting LSCs, but at the same time sparing HSCs, normal hematopoiesis, lymphoid cells and their progenitors [83].

**Preclinical studies.** Several preclinical studies have evaluated the efficacy of CAR-T therapy targeting the CLL-1 antigen in vitro and in vivo. Tashiro et al. reported the construction of anti-CLL-1 CAR-T cells, which selectively killed leukemic progenitor cells and their progeny [35]. The researchers used the CD3ζ signaling domain in combination with one co-stimulatory domain, 4-1BB (CLL-1.BBζ CAR-Ts). These cells showed significant cytotoxicity against HL60 and THP-1 cell lines, as well as proliferation under the conditions of CLL-1^+^ cell line stimulation. Similar results were obtained using primary AML samples. CLL-1.BBζ CAR-Ts have also been shown to inhibit leukemic colony formation. In order to confirm the anti-leukemic effects of CLL-1.BBζ CAR-Ts in vivo, the authors used the human xenograft mouse model of AML. Eight out of ten mice receiving the CAR-T cells had a lower leukemia burden than the control group, which was associated with longer survival time. One of the disadvantages of CAR-T cells targeting CLL-1 was their cytolytic activity against the mature cells of the myeloid lineage. The authors point out that the excessive toxicity of the CAR-T cells could be controlled, e.g., by introducing a safety switch based on inducible caspase 9 (iC9) [35]. Similar results were obtained by Wang et al., who constructed CAR-Ts consisting of CLL-1 scFv and two co-stimulatory domains—CD28 and 4-1BB. The obtained CAR-T cells lysed U937, HL60 cell lines and Raji cells with CLL-1 expression, as well as primary AML blasts. In in vivo studies on mice implanted with AML cells, the test group treated with CAR-T had significantly prolonged survival time and a significantly reduced number of tumor cells in peripheral blood [36]. Laborda et al. constructed anti-CLL-1 CAR-T cells and obtained similar results [84]. The anti-CLL-1 CAR-T cells completely eradicated tumors by day 90, while mice without treatment died within three weeks of tumor cell inoculation. In addition, CAR-T cells controlled disease relapse for 80 days after tumor regression. Researchers in in vitro studies investigated the anti-CLL-1 CAR-T cells activity on the mature cells of the myeloid line. CAR-T-induced cytolysis was found in neutrophils, but it was much lower than in HL60 cells, which might suggest that anti-CLL-1 CAR-T cells preferentially target tumor cells [84]. In all of the aforementioned in vitro and in vivo studies, there was no negative effect of anti-CLL-1 CAR-T cells on HSCs.

Some studies have investigated the possibility of increasing the effectiveness of anti-CLL-1 CAR-T cells. In one study, Atilla et al. compared the anti-CLL-1 properties of CAR-T cells with different configurations of spacers, transmembranes and endodomains. It turned out that CAR with CD28 as a co-stimulatory domain, a short hinge and/or CD8 endodomains showed the best proliferation, functional persistence and anti-tumor activity [85]. In another study, transgenic IL-15 was included in the anti-CLL-1 CAR-T construct to increase persistence and sustain the killing properties of T cells [86]. This solution resulted in greater anti-tumor activity in long-term cytotoxicity assays in vitro. However, in in vivo xenograft models of AML, the studied mice developed lethal CRS associated with very high levels of tumor necrosis factor α (TNFα). Therefore, the authors used anti-TNFα antibodies to avoid or delay toxicity, while maintaining the anti-leukemic effects of CAR-T cells. It is not known whether similar complications can occur in humans. The authors emphasize, however, that the combination of CAR-T cells with anti-TNFα antibodies and a security system, such as iC9, may prove to be an optimal solution [86]. Lin et al. investigated the use of anti-CLL-1 CAR-T cells in combination with programmed death receptor 1 (PD-1) silencing [87]. By binding to its ligands, PD-1 leads to T cell exhaustion. The authors created the third generation anti-CLL-1 CAR-Ts that effectively lyse CLL-1^+^ AML cells lines and primary AML blasts. After co-culturing THP-1 cells with effector T cells, an increased expression of PD-1 ligands was observed on THP-1 cells. Therefore, PD-1 silencing was additionally used, which resulted in an increase in the immunotherapeutic effect of anti-CLL-1 CAR-T cells. The same study also compared anti-CLL-1 CAR-T cells from healthy donors and patients, and showed that those from healthy donors had greater cytotoxicity against the THP-1 lineage [87].

**Clinical studies.** Due to the results of preclinical studies, clinical trials have been initiated and several attempts have been made to use anti-CLL-1 CAR-T cells in the treatment of AML in humans. Zhang et al. reported the use of anti-CLL-1 CAR-T cells therapy in a 10-year-old patient with secondary AML [88]. The authors created the fourth-generation CAR containing a CLL-1 specific fragment that was administered to a patient after prior lymphodepleting chemotherapy. The patient experienced CRS grades 1-2 and developed transient hypotension requiring fluid replacement. At the end of treatment, the patient achieved morphological CR and was minimal residual disease (MRD)-negative; however, CLL-1^+^ cells were not completely eliminated until six months after CAR-T cells infusion. Despite this, one dose of anti-CLL-1 CAR-T resulted in CR for ten months in this patient, suggesting that CLL-1-specific CAR-T cells might be a useful tool in AML therapy [88]. Another study evaluated the safety and efficacy of anti-CLL-1 CAR-T cells therapy in R/R-AML. The study involved four pediatric patients aged 7-9 years. Researchers constructed the fourth-generation CAR-T with CD28-CD27-CD3z signaling domains and iC9. All patients received a single dose of CAR-T cells after lymphodepleting chemotherapy so as to enhance the in vivo expansion of CAR-T cells. Three patients developed grade 1-2 CRS, but did not require intensive supportive care. One patient developed ICANS grade 1-2 requiring the administration of glucocorticoids for symptoms control. Three out of four patients achieved CR and MRD negativity. The fourth patient remained alive for five months [89]. In another clinical study, Jin et al. studied the effectiveness of anti-CLL-1 CAR-T cells in ten patients with R/R AML, whose average age was 43.5 years [90]. Following the administration of CAR-T cells, all patients developed CRS, six of them requiring corticosteroids and three of them additionally requiring tocilizumab. All patients had severe pancytopenia, nine had grade 3–4 agranulocytosis, seven had grade 3–4 anemia, and seven had grade 3–4 thrombocytopenia. As a result, two patients died from chronic infections. Of all patients, seven achieved CR/CRi and six were still alive at the last follow-up [90]. In another clinical study by Zhang et al., eight children with an average age of 12 years, suffering from R/R AML, received anti-CLL-1 CAR-T cells with endoplasmic domain 4-1BB. Grade 1–2 CRS was observed in all patients and no ICANS case was observed. The treatment effects were as follows: four patients had morphologic CR and MRD negativity, two patients had morphologic CR and MRD positivity, one patient had partial remission, and one patient remained stable for one month after starting the CLL-1 CAR-T cell therapy. Six patients received allo-HSCT; in one of them the disease relapsed after two months and the patient died of GvHD, in another case the disease relapsed after six months and the patient died of its progression. The remaining four patients who received HSCT were still alive and in CR at the last follow-up. Among the two patients who did not receive HSCT, the first maintained CR for 12 months, while the second one survived 3 months after the infusion of CAR-T cells [83]. 

Ma et al. reported the use of ant-CLL-1 CAR-T cells with PD-1 KO in two patients. Both patients had previously received anti-CD38 CAR-T cells therapy and underwent allo-HSCT, but in each case the disease relapsed after an initial remission. It was decided to start treatment with anti-CLL-1 CAR-T cells with PD-1 KO. Grade 1 CRS was observed in the first patient, but no ICANS was observed. White blood cells and neutrophils recovered after 23 days. On day 50 after CAR-T administration, allo-HSCT was performed. The first patient maintained MRD-negative CR for 8 months. The second one, after receiving anti-CLL-1 CAR-T cells infusion, suffered from grade 2 CRS, but no ICANS. That patient obtained CRi and MRD-negative CRi on day 28. These results prompted the authors to conduct a prospective clinical trial (NCT04884984) to confirm the effectiveness and safety of anti-CLL-1 CAR-T cells with PD-1 KO therapy [91]. 

In one recently published study, the results of the treatment of seven children with R/R AML with an average age of 8.4 years, using anti-CLL-1 CAR-T cells with different co-stimulatory domains, were reported. Four of them received CAR-T cells with CD28/CD27, and the remaining three with 4-1BB as co-stimulatory domains. Among the side effects, the most common was CRS, as all patients experienced it in grade 1 or 2. One patient developed ICANS grade 2, impaired liver function (which also occurred in one other patient), and grade 2 increased bilirubin level. Patients treated with 4-1BB CAR-T cells also developed pneumonia. Five patients achieved CR, and the overall response rate was 75% for patients with CD28/CD27 CAR-T cells, and 67% for patients with 4-1BB CAR-T cells. The 1-year survival was 57.1% among these patients, while at the time of publication of the results, only one patient was still alive. The remaining patients died due to disease progression, recurrence of leukemia or GvHD. The sole surviving patient also had a relapse at month 13 and, therefore, received a second dose of anti-CLL-1CAR-T cells as well as follow-up treatment with venetoclax, and remained leukemia-free [92]. 

### 2.6. Recruiting Clinical Trials

The results of preclinical studies, showing the effectiveness of CAR-T cells in eliminating AML cells, resulted in the commencement of clinical trials. Currently, 17 recruiting phase 1/2 clinical trials are being conducted, testing the possibility, effectiveness and safety of using anti-CD33, -CD123, -FLT3 or -CLL-1 CAR-T cells in AML therapy. For more information about currently recruiting clinical trials, see Table 2.

## 3. The Emerging Challenges and the Prospects for the Future

### 3.1. Adverse Events of CAR-T Cells Therapy

Common complications associated with the use of CAR-T cells include CRS and ICANS [6,110]. The cause of complications is one of the effector mechanisms of CAR-T cells, which, after binding the target antigen and being activated, release pro-inflammatory cytokines (e.g., interleukin 6—IL-6, interleukin 8, interleukin 10, IFN-γ, granulocyte macrophage colony-stimulating factor, macrophage inflammatory protein 1β, monocyte chemoattractant protein-1) [63,110,111]. The secreted cytokines contribute to the recruitment and activation of further cells of the immune system, intensifying the pro-inflammatory response, which causes the clinical symptoms of CRS. Although the pathogenesis of ICANS is not fully understood, it is believed that the increased concentration of cytokines is also responsible for the increased permeability of the blood–brain barrier. Pro-inflammatory cytokines and CAR-T cells penetrate through a leaky barrier into the central nervous system, causing the symptoms of neurotoxicity [63,110,111]. In the therapeutic management of CRS, tocilizumab (an antibody directed against the IL-6 receptor) and steroids (e.g., dexamethasone) are used [63,110,112]. Due to the poor penetration of tocilizumab into the CNS, its effectiveness in ICANS is limited [112]. In order to improve the safety of therapy, the iC9 suicide gene can be included in the CAR construct. In the event of significant toxicity and other adverse effects after the infusion of CAR-T cells, the activation of iC9 can be triggered by administering a neutral molecule to the patient, which will result in the elimination of therapeutic cells from the recipient’s body [47,113]. The disadvantage of this solution is the permanent loss of CAR-T cells. An alternative solution to this problem may be the regulation of CAR gene expression, thanks to which it will be possible to control the number of CARs expressed on CAR-T cells using biophysical or chemical inducers (e.g., Tet-ON technology enables the control of CAR gene expression depending on the dose of the administered tetracycline) [47]. The enhancement of the safety profile of CAR-T cells can also be obtained by overexpressing the runt-related transcription factor 3 (*RUNX3)* gene in them. This reduces the amount of pro-inflammatory cytokines released from CAR-T cells while maintaining their effective anti-cancer activity [114]. Work is also underway to obtain and test the anti-cancer efficacy of other types of immune system cells modified in order to express CAR, e.g., macrophages or natural killer (NK) cells. It has been shown that after activation, CAR-NK cells release a more favorable cytokine profile than CAR-T cells, which reduces the risk of developing CRS and ICANS when CAR-NK cells are used [111,115,116].

With a view to reducing the off-target toxicity of CAR-T cells, research is ongoing on the possibility of modulating the activity of CAR-T cells by reducing the affinity of the antigen-binding domain for the target molecule. In this case, a higher density of antigen on the tissue will be needed to activate CAR-T cells. This could result in the creation of CAR-T cells operating in the previously identified therapeutic window, in which CAR-T cells could destroy cancer cells with a higher expression of the target antigen without damaging healthy tissues with a lower expression [4,63,117]. The disadvantage of this approach is the potential for the downregulation of antigen expression by tumor cells, which may eventually lead to tumor escape from CAR-T surveillance. A solution to this problem may be a synergistic therapy combining the administration of CAR-T cells with agents that increase the expression of the target antigen on tumor cells. This can be achieved by the use of histone deacetylase inhibitors, which cause histone acetylation, or the use of methyltransferase inhibitors such as AZA or DAC, which cause DNA demethylation, ultimately leading to the activation of genes encoding target antigens for CAR-T cells [118,119]. Another possible way to increase the expression of the FLT3 antigen in AML is the use of a tyrosine kinase inhibitor, e.g., crenolanib, which blocks the activity of FLT3, resulting in the upregulation of FLT3 on leukemic cells [119]. The solution to the problem of the myeloablative activity of CAR-T cells in the case of AML therapy is the use of CAR-T cells for conditioning before allo-HSCT, or the administration of previously genetically modified hematopoietic cells to the patient during transplantation so as to eliminate the expression of the target antigen for CAR-T cells. In the first case, the myeloablative effect of CAR-T cells would be desirable. In the second case, the genetic modification of HSCs would lead to obtaining a tumor-specific antigen, which would result in proper BM regeneration in the presence of CAR-T cells in the recipient’s body [117]. In the case of a certain group of AML patients, the target antigen can be selected in such a way that the effector mechanism of CAR-T cells has a negligible effect on the process of myelopoiesis. Therefore, it has been reported that CD19 can be a target antigen in AML patients with t(8;21) [21]. Another possible way to increase the selectivity of CAR-T cells and reduce their unfavorable off-target effects is the use of logical gating, e.g., AND-gate, NOT-gate, IF-THEN-gate and IF-BETTER-gate [64]. In order to use AND-gate and NOT-gate, it is necessary to create Dual-CAR cells that co-express two independent CARs specific for different antigens. In the case of AND-gate, the Dual-CAR-T cell will activate and lyse only those cells that co-express both antigens. A prerequisite for the proper operation of Dual-CAR-T with AND-gate is the identification of two antigens that occur together on cancer cells, but are not present together on healthy tissues [64]. In the case of NOT-gate, the Dual-CAR-T cell expresses two different CARs, the first one transmitting an activation signal and the other one, based on the leukocyte immunoglobulin-like receptor-1protein with immunoreceptor tyrosine-based inhibitory motifs, an inhibitory signal (iCAR). Such a Dual-CAR-T cell will only become activated if the activation signaling CAR binds to its target antigen in the absence of the other target antigen that would bind to the iCAR. A prerequisite for the proper functioning of the NOT-gate is the identification of antigens, one of which is present on cancer cells and healthy tissues, and the other one is present on healthy tissues (with negligible expression on cancer cells). The first of the antigens would bind to the activation signaling CAR, and the second to the iCAR; therefore, the co-expression of antigens on healthy tissue would prevent the activation of NOT-gate Dual-CAR-T cells [64,120]. The use of the IF-THEN-gate enables the spatiotemporal regulation of CAR expression by using a synthetic Notch (SynNotch) receptor specific to the antigen present on tumor cells. Binding SynNotch to the target antigen (antigen I) induces the transient expression of CAR, specific for another antigen (antigen II) expressed by tumor cells, which leads to the activation of the CAR-T cell and the initiation of its effector mechanisms. In the presence of antigen II on healthy tissues, the off-target toxicity effect is limited by the transient expression of the receptor, regulated by SynNotch binding to antigen I present on cancer cells [64,121]. The use of IF-BETTER-gate also makes it possible to reduce the off-target toxicity effect. A CAR-T cell, apart from expressing CAR, expresses a chimeric co-stimulatory receptor (CCR) specific for an antigen other than CAR. CAR-T cell activation requires the presence of a significant amount of CAR target antigens or, in the case of a low amount of CAR target antigens, an additional combination of CCR with another antigen present on the tumor cell. Supplementing the CAR signal with CCR enhances the effector functions of CAR-T cells [64].

### 3.2. Immunosuppressive Tumor Microenvironment

Currently, studies are being conducted to increase the effectiveness of therapy using CAR-T cells, which is necessary due to the developing resistance to therapy in some cases resulting from the heterogeneous expression of antigens on cancer cells, the loss of the target antigen for CAR as the disease progresses, and the immunosuppressive TME, which can cause depletion and reduced viability in CAR-T cells [63,114]. TME consists of myeloid suppressor cells, tumor-associated macrophages and Treg-secreting cytokines that inhibit the anti-cancer response, and soluble immunosuppressive factors (transforming growth factor beta (TGF-β), interleukin 4, indoleamine 2,3-dioxygenase) [122]. The immune checkpoints PD-1 and CTLA-4 are also involved in inhibiting the anti-tumor response of CAR-T cells. For this reason, a synergistic therapy combining the administration of CAR-T cells with checkpoint inhibitors may contribute to overcoming the resistance of cancer cells to treatment, and in this way improve the functioning of CAR-T cells in TME [4,63]. Another option is to treat CAR-T cells with a bromodomain and extraterminal domain with the (*S*)-(+)-tert-Butyl 2-(4-(4-chlorophenyl)-2,3,9-trimethyl-6*H*-thieno(3,2-f)(1,2,4)triazolo(4,3-a)(1,4)diazepin-6-yl)acetate (JQ1) inhibitor, which reduces the expression of PD-1 and T cell immunoglobulin and mucin domain-containing protein 3 (TIM-3) on T lymphocytes. An additional advantage of JQ1 is that it reduces the expression of PD-1L on cancer cells [123]. Another approach is to use the clustered regularly interspaced short palindromic repeats (CRISPR)/CRISPR-associated protein 9 (Cas9) technique in the production of CAR-T cells to remove genes encoding inhibitory molecules (CTLA-4, PD-1, lymphocyte-activation gene 3), TGF-β receptor or E2 ligase Casitas B-lineage lymphoma-b, which upregulate PD-1 and TIM-3 expression, thanks to which CAR-T cells could overcome the immunosuppressive effect of TME [117,124]. The increased expression of thymocyte selection-associated high-mobility group-box family members 1 and 2 (*TOX* and *TOX2)* transcription factors, as well as members of the nuclear receptor subfamily 4A (*NR4A*), has also been shown to induce T cell depletion by upregulating genes encoding inhibitory molecules. In studies on tumor-inoculated mice, it was shown that CAR-T cells deficient in both *TOX* and *TOX2,* or with triple KO of *NR4A1*, *NR4A2*, and *NR4A3,* had a greater anti-cancer activity and improved animal survival; therefore, targeting the *TOX*/*NR4A* axis may reduce the depletion of CAR-T cells in TME [124,125]. The function of CAR-T cells is also affected by their quality and innate phenotype. It is postulated to transform CAR-T cells into central memory T cells or T cells similar to stem cells, which will increase their endurance and prolong their presence in the body [122].

### 3.3. Limitations in the Preparation of CAR-T Cells

Due to the risk of developing GvHD, each patient receives a CAR-T preparation prepared from autologous T lymphocytes. The process of preparing CAR-T cells is time-consuming and expensive. First, a patient qualified for CAR-T therapy undergoes leukapheresis; then, T lymphocytes are isolated from the isolated fraction of peripheral blood mononuclear cells. The cells are then activated ex vivo through their endogenous TCR-CD3 complex. The next step is the transduction of CAR genes into autologous T lymphocytes using viruses (lentiviral/retroviral transduction) or the mRNA electroporation method. After expansion, CAR-T cells are administered to the patient [64,111,126]. In addition to the high cost of preparing CAR-T cells, the effectiveness of CAR-T cells depends on the quality of the T cells collected from the patient. In addition, it can be difficult to obtain adequate numbers of T cells from lymphopenia patients. Research is, therefore, underway to develop CAR-expressing allogeneic T cells (UCAR-T) that would not induce GvHD in the recipient. This is possible thanks to the use of CRISPR/Cas 9 technology to remove genes encoding endogenous TCR and HLA class I [127,128]. However, the lack of expression of HLA class I molecules may lead to the activation of NK cells of the recipient and the rejection of UCAR-T by NK cells [129]. Constructing a fusion protein beta-2-microglobulin and HLA-E and introducing it into UCAR-T can prevent their rejection by NK cells [130]. Obtaining allogeneic CAR-T cells from induced pluripotent stem cells will lower the production costs of the preparation, and may lead to the creation of ready-made, universal UCAR-T drugs available immediately, “off the shelf” [127]. The next step to creating a universal drug based on UCAR-T is the use of UniCAR technology. UniCAR cells are devoid of cytotoxic activity until TMs are administered, which targets them to the tumor cell and enables the activation of cytotoxic mechanisms; therefore, the elimination of TMs from the body in the event of side effects prevents the activation of UniCAR cells. The advantage of UniCAR cells over CAR cells is the ability to target the same UniCAR cells to different tumor antigens by modifying only the target module, and their increased safety profile [46,47]. The modification of UniCAR cells is the RevCAR technology, in which the size of the gene encoding the CAR construct is reduced by swapping the locations of the peptide epitope and the peptide epitope-binding domain [68]. In order to improve the controllability of UniCar and RevCAR cells, split, universal, and programmable (SUPRA) CAR system cells were created. SUPRA CAR consists of two parts: a universal receptor with a leucine zipper adapter (zipCAR), expressed on cells, and a separate scFv with a leucine zipper adapter (zipFv) molecule, directed against the target antigen. A leucine zipper (AZip), linked to scFv, can link to the cognate a leucine zipper (BZip) present on the zipCAR. The zipFv binding to the target antigen and dimerizing with the zipCAR results in the activation of the SUPRA CAR cell. Similar to UniCAR and RevCAR, SUPRA CAR cells can be targeted to different tumor antigens by modifying only the zipFv. In addition, SUPRA CAR cells have an increased safety profile compared to other CARs cells because their effector functions can be modulated by changing the configuration of the leucine lock [47,128]. Further modification of the SUPRA CAR cells enables the use of logic gating [47]. The use of the UniCAR, RevCAR or SUPRA CAR technology will reduce the production costs of a preparation based on UCAR-T cells, because only the antigen against which the TM or zipFv is directed will need to be modified. A comparison of the constructions of UniCAR, RevCAR and SUPRA CAR is shown in Figure 3.

## 4. Conclusions

Despite some promising results from preclinical/clinical studies, some challenges must be overcome for the widespread use of CAR-T cells in AML therapy and the registration of a drug based on this technology. Due to the presence of CAR-T target antigens on normal cells, it is necessary to use either an anti-myeloablative strategy or CAR-T cells for the depletion of HSCs. In addition, because of adverse events, it is mandatory that the therapy with the use of CAR-T cells should contain a safety switch. It is also essential to extend the persistence of CAR-T cells in the body, as well as improve their functioning in TME, so as to increase their efficiency. For some patients, the formation of CAR-T cells could be impossible due to the failure of aphaeresis; additionally, because of the long production time of the drug, some patients might die before the administration of CAR-T cells. For this reason, research contributing to the creation of universal allogenic CAR-T cells is still needed. Its positive outcomes might translate to a reduction in the time needed to prepare CAR-T cells, and also a decrease in the cost of their production so as to make them available to a larger number of patients.

## Figures and Tables

**Table 1:** Possible antigen targets for CAR-T cells in AML.

Antigen	Expression on AML Leukemic Cells	Expression on LSCs	Expression on Healthy Hematopoietic Cells	References
CD33 Siglec-3	90–99%	+	myeloid progenitor cells, unipotent colony-forming cells, maturing granulocytes and monocytes	[25,26,27,28]
CD123 IL-3Rα	50–78%	+	low level/no expression on HSCs, monocytes, plasmoid dendritic cells, basophils	[29,30,31,32,33]
FLT3 CD135	54–92%	+	HSCs, myeloid progenitor cells	[24,34]
CLL-1	78–92%	+	granulocytes, monocytes and their progenitors	[35,36]

AML—acute myeloid leukemia, LSCs—leukemic stem cells, CD33 (Siglec-3)—sialic acid binding Ig-like lectin 3, CD123 (IL-3Rα)—α chain of the interleukin-3 receptor, FLT3 (CD135)—fms-like tyrosine kinase 3, CLL-1—C-type lectin-like molecule-1, “+”—the presence of expression on LSCs HSCs—hematopoietic stem cells.

**Table 2:** Recruiting clinical trials testing the use of anti-CD33, -CD123, -FLT3 or -CLL-1 CAR-T cells in acute myeloid leukemia therapy.

Drug	ClinicalTrials.gov Identifier	Phase of Clinical Study	Estimated Numbers of Patients	Studied Patient Population	Dosage	References
Anti-CD33 CAR-T cells	NCT04835519	Phase 1, Phase 2	25	Patients with R/R CD33^+^ AML, 1–70 y.o.	Two dose levels—DL1: 5 × 10^6^ cells/kg DL2: 1 × 10^6^ cells/kg	[93]
SC-DARIC33 (anti-CD33 CAR-T cells)	NCT05105152	Phase 1	18	Patients with CD33^+^ AML, up to 30 y.o.	-	[94]
PRGN-3006 T Cells (anti-CD33 CAR-T cells)	NCT03927261	Phase 1	88	Patients with R/R AML or higher risk MDS, above 18 y.o.	-	[95]
Anti-CD123 CAR-T cells	NCT04318678	Phase 1	32	Patients with R/R CD123^+^ disease, up to 21 y.o.	Four dose levels—DL1: 3 × 10^5^ cells/kg DL2: 1 × 10^6^ cells/kg DL3: 3 × 10^6^ cells/kg DL4: 1 × 10^7^ cells/kg	[96]
Anti-CD123 CAR-T cells	NCT04272125	Phase 1, Phase 2	40	Patients with R/R AML, 3–75 y.o.	-	[97]
UCART123v1.2 (Allogeneic Engineered T cells Expressing Anti-CD123 CAR)	NCT03190278	Phase 1	65	Patients with CD123^+^ relapsed or primary refractory AML, 18–65 y.o.	-	[98]
TAA05 (anti-FLT3 CAR-T cells)	NCT05432401	Early Phase 1	18	Patients with FLT3^+^ R/R AML, 18–70 y.o.	Three dose levels—DL1: 1 × 10^8^ cells, DL2: 2 × 10^8^ cells, DL3: 4 × 10^8^ cells	[99]
TAA05 (anti-FLT3 CAR-T cells)	NCT05445011	Phase 1	12	Patients with FLT3^+^ R/R AML, 18–70 y.o.	Three dose levels—DL1: 1 × 10^8^ cells, DL2: 2 × 10^8^ cells, DL3: 4 × 10^8^ cells	[100]
Anti-FLT3 CAR-T	NCT05023707	Phase 1, Phase 2	5	Patients with FLT3^+^ R/R AML, 16–65 y.o.	-	[101]
TAA05 (anti-FLT3 CAR-T cells)	NCT05017883	Not applicable	5	Patients with FLT3^+^ R/R AML, 18–70 y.o.	-	[102]
Anti-CLL-1 CAR-T cells	NCT05252572	Early Phase 1	36	Patients diagnosed with CLL-1^+^ R/R AML at any age.	2–8 × 10^6^ cells/kg	[103]
Anti-CLL-1 CAR-T cells	NCT04923919	Early Phase 1	100	Patient with CLL-1^+^ R/R AML, 2–75 y.o.	-	[104]
Anti-CLL-1 CAR-T cells	NCT04219163	Phase 1	18	Patients with primary R/R AML, up to 75 y.o.	Three dose levels—DL1: 1 × 10^7^ cells/m^2^; DL2: 3 × 10^7^ cells/m^2^; DL3: 1 × 10^8^ cells/m^2^;	[105]
KITE-222 (anti-CLL-1 CAR-T cells)	NCT04789408	Phase 1	40	Patients with R/R AML, older than 18 y.o.	-	[106]
Anti-CLL-1 CAR-T cells with PD-1 KO	NCT04884984	Phase 1, Phase 2	20	Patients with CLL-1^+^ R/R AML, 6–65 y.o.	5–20 × 10^6^ cells/kg	[91,107]
CLL-1, CD33 and/or CD123-specific CAR-T cells	NCT04010877	Phase 1, Phase 2	10	Patients with AML with expression of CLL-1, CD123 and/or CD33, 0.5–75 y.o.	-	[108]
Dual CD33/CLL-1 CAR-T	NCT05248685	Phase 1	20	Patients with AML with co-expression of tumor surface antigens CD33 and CLL-1, 1–70 y.o.	Starting dose 1: 1 × 10^6^ cells/kg; Dose 2: 5 × 10^6^ cells/kg	[109]

AML—acute myeloid leukemia, R/R—relapsed/refractory, y.o.—years old, DL—dose level, MDS—myelodysplastic syndrome, CD33 (Siglec-3)—sialic acid binding Ig-like lectin 3, CD123 (IL-3Rα)—α chain of the interleukin-3 receptor, FLT3 (CD135)—fms-like tyrosine kinase 3, CLL-1—C-type lectin-like molecule-1.

## Data Availability

Not applicable.
